# Block-And-Lock: New Horizons for a Cure for HIV-1

**DOI:** 10.3390/v12121443

**Published:** 2020-12-15

**Authors:** Ines Moranguinho, Susana T. Valente

**Affiliations:** The Scripps Research Institute, Department of Immunology and Microbiology, 130 Scripps Way, Jupiter, FL 33458, USA; moranguinho.ines@gmail.com

**Keywords:** antiretroviral therapy, HIV-1 latency, HIV-1 reservoir, functional cure, block-and-lock

## Abstract

HIV-1/AIDS remains a global public health problem. The world health organization (WHO) reported at the end of 2019 that 38 million people were living with HIV-1 worldwide, of which only 67% were accessing antiretroviral therapy (ART). Despite great success in the clinical management of HIV-1 infection, ART does not eliminate the virus from the host genome. Instead, HIV-1 remains latent as a viral reservoir in any tissue containing resting memory CD4^+^ T cells. The elimination of these residual proviruses that can reseed full-blown infection upon treatment interruption remains the major barrier towards curing HIV-1. Novel approaches have recently been developed to excise or disrupt the virus from the host cells (e.g., gene editing with the CRISPR-Cas system) to permanently shut off transcription of the virus (block-and-lock and RNA interference strategies), or to reactivate the virus from cell reservoirs so that it can be eliminated by the immune system or cytopathic effects (shock-and-kill strategy). Here, we will review each of these approaches, with the major focus placed on the block-and-lock strategy.

## 1. Introduction

As of the end of 2019, 38 million people worldwide were living with HIV-1 and 1.7 million of those were newly diagnosed [1]. Antiretroviral therapy (ART) has turned a disease that was once a death sentence, into a chronically manageable condition. ART reduces HIV-1 replication to undetectable levels in the blood and tissues of individuals living with HIV-1, preventing the spread of the virus. Although very efficient, ART is not curative, and the HIV-1 provirus remains integrated into the host genome, mainly in resting memory CD4^+^ T cells [2]. Upon ART interruption, this stable viral reservoir can reinitiate rebound viremia within weeks [3], and therefore elimination of this reservoir is critically important to achieve a sterilizing cure [4]. ART is a lifelong commitment and curative strategies are urgently needed. Of note, long-term toxicity and persistent immune activation, specifically monocyte activation, are associated with lifelong ART [5,6,7]. The prevalence of acquired drug resistance in treated individuals and the spread of drug-resistant viruses also becomes increasingly more prominent with lifelong ART [8]. Thus, the current development of long-acting injectable formulations [9] seeks to improve adherence and effectiveness of HIV-1 treatment and prevention [10].

Several strategies for an HIV-1 cure have been explored. These mainly focus on the removal of the provirus from the host cell genome, elimination of cells containing the provirus, or blocking important HIV-1 or host factors required for replication [11]. Editing the CCR5 gene has been one of the main focuses of gene therapy strategies to generate HIV-1-resistant cells [12]. This approach has been motivated by the successful cure of Timothy Ray Brown, the so-called Berlin patient. Timothy Brown was diagnosed with HIV-1 in 1995 and is known as the first person to be cured of HIV-1. He received two allogeneic hematopoietic stem-cell transplantations (HSCT) for treatment of acute myeloid leukemia from a donor with a homozygous mutation in the gene of the HIV-1 coreceptor CCR5 (CCR5Δ32/Δ32) [13]. Sadly, he recently passed away (29 September 2020) from returning leukemia, an immense loss as he was a tireless champion and advocate for an HIV-1 cure. In 2019, another individual living with HIV-1, known as the London patient, underwent a single allogeneic HSCT with a homozygous CCR5Δ32 mutation to treat Hodgkin’s lymphoma, and HIV-1 remission has been maintained so far [14]. Even though these two individuals raised the hope of achieving an HIV-1 cure, a practical and accessible cure for everyone is still far beyond reach. In recent years, attempts have been made to develop a functional cure, aimed at the durable control of HIV-1 replication in the absence of treatment. This concept was based on the finding of rare individuals, termed post-treatment controllers (PTCs), who maintained viral replication suppressed for months or years after treatment interruption. Additionally, rare individuals called “elite” controllers can maintain the viral load below the limit of detection for years in the absence of ART [15]. Recently, Jiang et al., reported that proviruses from elite controllers are in a deeper state of latency when compared to proviruses found in individuals under suppressive ART [16]. The strong viral transcriptional suppression observed in elite controllers highlights the possibility of a functional cure through block-and-lock approaches to achieve an ART-free remission [17]. Whether the goal is a sterilizing cure with full elimination of HIV-1 provirus from reservoirs or a functional cure with immunological control of persistent HIV-1, in depth comprehension of the mechanisms of HIV-1 latency and transcription are required [18]. Here, we will review these mechanisms as well as existing block-and-lock approaches to silence proviral expression.

## 2. HIV-1 Latency

The latent reservoir is established within days of HIV-1 infection and is composed mainly of long-lived memory CD4^+^ T cells that harbor integrated latent provirus. These cells containing latent proviruses persist in part via homeostatic or antigen-driven proliferation [19,20]. The latent reservoir decays very slowly in individuals on ART, and it has been estimated that it would take over 73 years to eradicate this reservoir, a very grim prospect [4,21,22]. There is, however, growing evidence that ART initiation very soon after HIV-1 infection may be effective in reducing the HIV-1 reservoir size [23]. Recently, Ndhlovu et al., suggested that ART initiation immediately after HIV-1 diagnosis also preserves immune function with enhanced functionality of HIV-1-specific CD4^+^ and CD8^+^ T cells [24]. Of note, an ongoing clinical trial (NCT02859558) seeks to evaluate the efficacy of early ART initiation on HIV-1 persistence and HIV-1-specific immune responses.

Although still unclear, one of the most accepted explanations for latency establishment is the return of infected activated CD4^+^ T cells to a resting memory state, unfavorable to HIV-1 replication [4,22]. Infection of activated CD4^+^ T cells usually results in cell death; however, when infected cells survive long enough, they revert to a resting memory state, estimated at 0.01–100 per 10^6^ CD4^+^ T cells, with minimal proviral gene expression [22]. The low frequency of latently infected cells rends the quantification of the reservoir extremely difficult. Thus, an accurate and scalable assay for latent reservoir measurement is urgently needed. The quantitative viral outgrowth assay (QVOA) was first used in the mid-1990s and measures the frequency of resting CD4^+^ T cells harboring replication-competent proviruses [25]. However, this method underestimates the true size of latent reservoir size because of the presence of non-induced proviruses by a single round of T cell activation [26]. It should be noted that over 90% of proviruses are replication-defective [27]. On the other hand, PCR-based assays that measure total HIV-1 proviral DNA by targeting conserved regions of the HIV-1 genome overestimate the reservoir size, since they do not distinguish between defective and replication-competent viruses (reviewed in Massanela et al. [28]). Overall, QVOA is considered the gold standard since it provides a minimal estimate of reservoir size [29].

The environment surrounding the HIV-1 integration site is an important factor contributing to the establishment of HIV-1 latency [30]. HIV-1 proviral DNA integration can occur in genomic regions where access to transcription factors (TFs) and RNA polymerase II (RNAPII) is abundant or restricted [31]. HIV-1 preferentially integrates into actively transcribed regions of the host chromatin, and thus the chromatin environment plays a key role in HIV-1 transcriptional regulation (discussed in Section 3) [32]. The absence of viral RNA and protein expression limits the ability of the immune system to distinguish between latently infected cells and uninfected cells [4,33]. Therefore, efforts have been made to identify biomarkers that could be used to identify HIV-1 latently infected cells. CD2 and CD32a were proposed as markers [11,34,35]; however, either by the low abundance of these cells or by technical challenges, their significance is still debated.

The HIV-1 reservoir is not static, and replenishment of the latent reservoir may occur by residual levels of viral replication (blips) and clonal expansion of infected cells over time [36,37]. The contribution of antigen-induced or homeostatic proliferation of infected cells are factors that contribute to this clonal expansion [38]. As in a vicious cycle, the immune activation of T cells, driven by viral antigens produced by either intact proviruses or by the approximately >90% defective proviruses, also contributes to the replenishment of the latent reservoir [30,37]. On the other hand, T cell activation also provides a pool of susceptible target cells for infection.

Eliminating or controlling the latent reservoir has become a research priority. It is thus of extreme importance to develop improved assays to identify and measure the HIV-1 reservoir size to facilitate the efficacy of HIV-1 functional cure approaches.

## 3. HIV-1 Transcription

HIV-1 transcription depends on a complex combination of viral and cellular factors to maximize viral gene expression. Additionally, the chromatin environment surrounding the HIV-1 promoter is highly relevant and can dictate the transcriptional state of the provirus [39]. Transcription is driven by the promoter, the 5’-long terminal repeat (5´-LTR). This promoter contains multiple binding sites for host transcriptional factors that help recruit RNA polymerase II (RNAPII) and chromatin modifiers to enhance its own transcription [40,41]. Tat, a small nuclear viral protein, is the main player in viral transcription regulation by promoting RNA polymerase II (RNAPII) transcription elongation from the LTR promoter [42].

### Tat-Mediated HIV-1 Transcription

**Figure 1:**
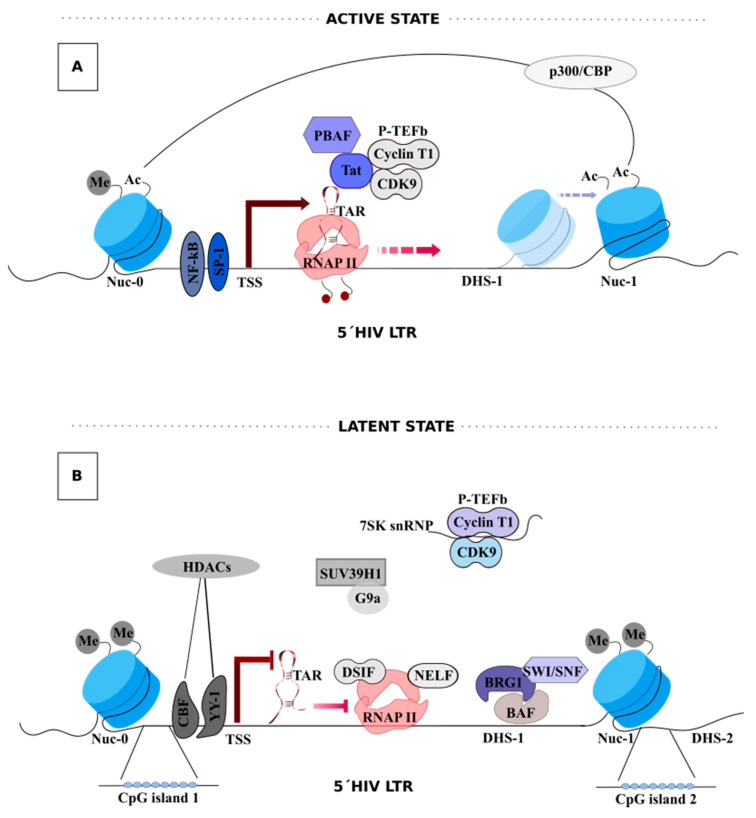
Regulation of HIV transcription. (**A**) Tat-mediated HIV-1 transcription. The transition from transcriptional initiation to elongation implies the replacement of transcriptional repressors by activators, including NF-κB, SP-1, and histone acetyltransferases (HATs), such as p300/CREB-binding protein (CBP). HATs promote open chromatin and recruitment of polybromo-associated factor (PBAF). PBAF repositions Nuc-1 further downstream of the transcription start site (TSS), enabling efficient transcriptional elongation. Furthermore, the secondary structure of the nascent TAR RNA is also accessible for the biding of Tat protein and further transcription activation. P-TEFb-associated CDK9 phosphorylates the CTD of RNAPII at serine 2, enabling the production of full-length HIV-1 transcripts, which are spliced to produced Tat and an active transcription elongation is established. A Tat-dependent positive feedback loop is generated by increasing HIV-1 transcription and, consequently, exponential replication. (**B**) Establishment of HIV-1 latency. Several proteins are implicated in the establishment of HIV-1 latency. These include transcription factors such as YY-1 and CBF, enabling the recruitment of histone deacetylases (HDACs). HDACs remove acetyl groups from core histones, namely, at Nuc-1, restricting the accessibility of positive transcription factors to the promoter, promoting viral latency. Histone methyltransferases (HMTs) involved in the establishment of HIV-1 latency include SUV39H1 and G9a, which are involved in Lys9 trimethylation of histone H3 (H3K9me3) and H3K9 dimethylation (H3K9me2), respectively. Two factors, DSIF and NELF, co-operate to pause RNAPII. Repressive factors such as BAF favor HIV-1 latency by positioning Nuc-1 in an energetically unfavorable position downstream of the TSS. Finally, P-TEFb is inactive through its association with the 7SK snRNP, preventing the transition from transcriptional initiation to elongation.

The switch from latent to active transcription is mostly dependent on the powerful feedback mechanism fueled by the early HIV-1-encoded Tat protein (Figure 1A) [43]. In the absence of Tat, nuclear factor (NF)-κB, the nuclear factor for activated T cells (NFATc), specificity protein 1 (Sp1), and the TATA-binding protein (TBP) enable RNAPII initiation at the 5´-LTR through the recruitment of the pre-initiation complex (PIC) and RNAPII. Shortly, after trans-activation response element (TAR) RNA, RNAPII pauses and accumulates at Nuc-1, producing short abortive transcripts [44,45]. HIV-1 TAR RNA is a highly folded stem-loop structure present at the 5´end of all nascent spliced and unspliced HIV-1 mRNAs and is synthesized by RNAPII just before pausing [46]. Among several host factors, the 5,6-Dichloro-1-β-d-ribofuranosylbenzimidazole (DRB) sensitivity-inducing factor (DSIF) and negative elongation factor (NELF) function co-operatively to mediate RNAPII pausing [47]. The release of RNAPII from Nuc-1 and consequently RNAPII elongation is mediated through the cellular positive transcriptional elongation factor b(P-TEFb), a heterodimer kinase composed of CDK9 and cyclin T1, which is thought to be recruited to the LTR by BRD4 and NF-κB. P-TEFb-associated CDK9 then phosphorylates the two repressive factors DSIF and NELF, which results in NELF detachment from the PIC, and DSIF conversion into a positive elongation factor. P-TEFb also phosphorylates the carboxyl-terminal domain (CTD) of RNAPII at serine 2, enabling the production of full-length HIV-1 transcripts [48], and eventually Tat protein [49,50,51,52]. Tat amplifies viral transcription by enhancing recruitment of P-TEFb to the TAR RNA, as well as recruitment of other components of the super elongation complex (SEC), to promote RNAPII transcriptional elongation [53,54,55]. Whether Tat binds to TAR alone or as a complex with P-TEFb is still a matter of debate [46].

In sum, when a Tat-dependent positive feedback loop is generated, it increases HIV-1 transcription and, consequently, leads to exponential replication. Thus, due to Tat’s crucial role in the production of full-length HIV-1 transcripts, it is inevitably a fantastic therapeutic target. 3.2. Epigenetic Regulation

Nucleosomes (Nuc) are precisely positioned at the promoter independently of the integration site. Nuc-0, Nuc-1, and Nuc-2 are separated by DNase hypersensitive regions 1 and 2 (DHS-1 and -2) [56,57,58]. Nuc-1, located immediately downstream of the transcription start site (TSS), is an essential regulator of HIV-1 transcription, since epigenetic modifications in this region determine the accessibility of TFs and RNAPII to the proviral DNA.

Chromatin remodeling complexes with ATPase domains and histone N-terminal tails regulate/read epigenetic modifications that alter the accessibility to the DNA embedded within nucleosomes [59]. Chromatin remodeling complexes actively add (writers), remove (erasers), or recognize (readers) post-translation modifications (PTMs) on histones. BAF (BRG1- or HBRM-associated factor) and PBAF (polybromo-associated factor) are ATP-dependent SWI/SNF chromatin remodeling complexes with opposite effects on chromatin structure [60]. BAF represses transcription, favoring HIV-1 latency, by positioning Nuc-1 in an energetically unfavorable position downstream of the TSS [61]. On the contrary, PBAF is recruited by acetylated Tat, and using energy from ATP hydrolysis actively repositions Nuc-1, enabling efficient transcriptional elongation [59,61,62]. Deacetylation and methylation of histone N-terminal tails also regulate HIV-1 transcription given their effect on nucleosome stability [63]. Deacetylation relies predominantly on the activity of histone deacetylases (HDACs). Host transcription factors, such as ying-yang 1 (YY1), late SV40 factor (LSF), C-repeat binding-factor 1 (CBF-1), and NF-κB p50, enable the recruitment of HDACs to the LTR, resulting in the removal of acetyl groups from core histones, namely, at Nuc-1, restricting the accessibility of positive transcription factors to the promoter, and consequently promoting viral latency [64,65]. Thus, LSF and YY1, together, allow for the recruitment of HDACs downstream of TSS [66].

Histone methyltransferases (HMTs), such as the suppressor of variegation 3–9 homolog 1 (Suv39H1) and G9a, favor the entry into latency and are involved in Lys9 trimethylation of histone H3 (H3K9me3) and H3K9 dimethylation (H3K9me2), respectively [67,68].

The role of CpG methylation of the HIV-1 promoter in the regulation of HIV-1 transcription, through the recruitment of repressor proteins or restrictive access of transcription factors to the target DNA [69,70], is still debated. Some in vitro studies demonstrated that methylation of the HIV-1 promoter strongly stabilizes HIV-1 latency in cultured HIV-1-infected cells [69,71,72]. However, these results are not concordant with some in vivo studies. J. Blazkova et al. revealed low levels of methylation at the HIV-1 promoters in the latent reservoir of ART-adherent aviremic individuals living with HIV-1 [73]. By contrast, J. Palacios et al. showed that latent proviruses in long-term non-progressors and elite controllers exhibit some degree of HIV-1 promoter methylation as compared to progressors with ART suppressed viremia, which exhibit no methylation [74]. Thus, additional studies are needed to clearly define the exact role played by CpGmethylation in the regulation of HIV-1 transcription.

The bromodomain-containing protein 4 (BRD4) is another important player that mediates interaction with acetylated histones and is involved in both positive and negative regulation of HIV-1 transcription [50,75]. BRD4 encodes two isoforms, a long isoform (BRD4L) and a short isoform (BRD4S) [76]. BRD4L contains a positive transcription elongation factor complex (P-TEFb) interacting domain (PID), which activates basal HIV-1 transcription in the absence of Tat [50,77,78].

Finally, reactivation of viral transcription from latency involves histone acetylation by histone acetyltransferases (HATs), such as CREB-binding protein (CBP) and p300, p300/CBP-associated factors, and the histone acetyltransferase hGCN5, to create an accessible chromatin environment for transcription to occur [62,79,80,81]. Together, the availability of TFs, the chromatin organization, and the epigenetic environment of HIV-1 promoter are important regulatory components for the establishment of a silenced provirus (Figure 1B).

## 4. Latency Reversal Agents

**Figure 2:**
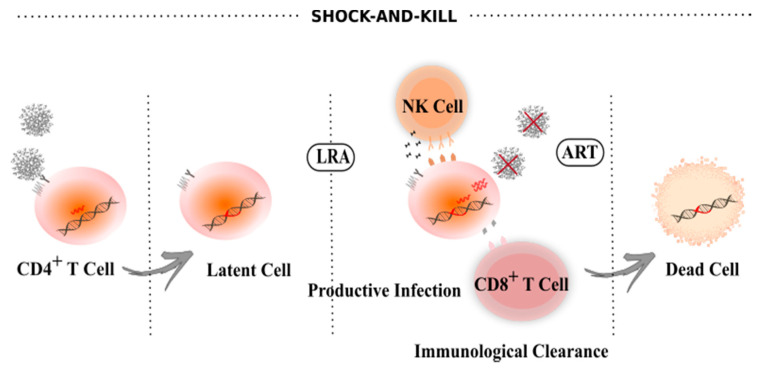
Shock-and-kill approach to eradicate HIV-1 from latently infected cells. During HIV-1 infection, the majority of CD4^+^ T cells are eliminated through cytopathic effects; the few surviving cells revert to a resting memory state harboring latent proviruses. The shock-and-kill approach uses latency reversal agents (LRA) to increase HIV-1 transcription/replication and virions production. This reactivation leads to the elimination of infected cells by cell cytolysis or immune clearance, and simultaneously the remaining viruses are blocked from novel infections by ART.

One strategy to achieve HIV-1 eradication is to purge all proviruses capable of replication from the latent reservoir. In theory, this could be achieved by using latency reversing agents (LRAs) to promote virus transcription and virion formation, which would render the infected cell susceptible to cytolysis and recognition/clearance by the immune system, while novel rounds of infection are prevented by ART [33,82]. This strategy is known as shock-and-kill (Figure 2) [83,84,85]. As of today, more than 300 molecules have been evaluated in vitro to reactivate latent HIV-1, including epigenetic, chromatin, signaling, and transcription modulators [86,87]. The chromatin-modifying agents, HDAC inhibitors (e.g., Vorinostat, Romidepsin and Panobinostat), are the most characterized class of LRAs explored in shock-and-kill approaches [86]. Three clinical trials using vorinostat clearly demonstrated an increase in HIV-1 RNA in resting CD4^+^ T cells; however, this was insufficient for robust protein production and did not have a meaningful impact on the size of the HIV-1 reservoir [88,89,90]. Nevertheless, the first Vorinostat clinical trial reported by Archin N. et al. [88] proved the concept that proviruses from HIV-1 reservoirs can be reactivated in humans, promoting research for novel LRAs. Romidepsin and Panobinostat, which showed higher reactivation potential in vitro, underwent clinical trials, but, similar to vorinostat, no significant differences were observed on the clearance or size of the HIV-1 reservoir [91,92,93]. Another class of LRAs, protein kinase C (PKC) agonists, are also undergoing extensive research in vitro and in clinical trials; however, these trigger NF-κB activation, which may activate T cells in addition to HIV-1, quite an undesirable effect [94]. Bryostatin-1 and ingenol 3,20-dibenzoate reversed latency more potently when compared to HDAC inhibitors in ex vivo assays (using cells from individuals living with HIV) [95,96]. So far though, despite a slight increase in HIV-1 latency reversal, these molecules alone are unable to significantly reduce the size of the latent reservoir. The combinatory use of LRAs with synergic effects is currently an active area of research [97,98,99]. Using latently infected cell lines, Laird G. et al. demonstrated that combining PKC agonists with the bromodomain inhibitor JQ1 or HDAC inhibitors robustly reversed latency without inducing proinflammatory cytokine production [98]. An ongoing clinical trial (NCT03525730) is evaluating the synergistic effect between Pyrimethamine, a BAF chromatin remodeling complex inhibitor, and Valproic acid, an HDAC inhibitor, on reducing the HIV-1 reservoir in ART-suppressed individuals. Of note, some in vitro studies suggest that the negative impact of LRAs on CD8^+^ T cell function could compromise the clearance of reactivated cells, review by Clutton G. et al. [100]. Thus, combinatorial approaches using more than one LRA may have beneficial effects as individual drug doses may be reduced, lowering the toxic effect on CD8^+^ T cell function.

For efficient clearance of latently infected cells, LRA-mediated viral protein expression must be sufficiently high for its consequent presentation by major histocompatibility class I (MHC-I) and recognition by functional HIV-1-specific CD8^+^ T cells [100]. As such, strategies to increase anti-HIV-1 immune responses are being explored in combination with LRAs to purge the reservoir. The first clinical trial with feasible combined strategies used Romidepsin boost with a T cell vaccine which resulted in a small reduction in HIV-1 DNA [101]. Despite promising results in tissue culture, existing LRAs are not potent enough to fully reactivate all HIV-1 proviruses hidden in a variety of cellular and tissue reservoirs [82,102,103]. Moreover, LRAs do not have the same impact on all infected cells, suggesting that cell susceptibility to LRAs may result from the interplay between viral and host factors [104]. Additionally, insufficient cytotoxic T cell responses result in minimal effects on the reservoir size [105]. Of note, some studies suggest that the use of LRAs could increase immune activation, leading to harmful inflammatory responses [106]. With respect to this, Gama L. et al. observed an increase in immune activation markers (CCL2 and neopterin) and neuronal damage in the central nervous system (CNS) in SIV-infected pigtailed macaques treated with LRAs (ingenol-B and vorinostat) [106]. Despite CNS-penetrant suppressive ART, they found 10 times higher viral load in cerebrospinal fluid than plasma, which forced euthanasia of the animals.

Altogether, reactivation from latency faces numerous challenges, namely, (1) heterogeneous reservoir of infected CD4^+^ T cells in various tissue compartments, (2) different clonal proliferation rates, and (3) the presence of intact or defective viruses [20,37,107]. Therefore, strategies that are not dependent on reactivating HIV-1 transcription have also been explored.

## 5. Block-And-Lock

HIV-1 eradication, which entails the full elimination of the viral reservoir is a tall order and has not been achieved so far, thus prompting additional efforts geared towards a functional cure. A functional cure is defined as the permanent suppression of HIV-1 transcription, with undetectable virus replication, normal CD4^+^ T cell counts, no disease progression and no viral transmission in the absence of treatment, despite the presence of integrated proviruses. With this approach, integrated HIV DNA is not fully eradicated but viral transcription is absent or low enough that any occurring viral production can be cleared by the immune system [108,109]. Affordability, long-lasting effects, and applicability in resource-poor settings, are features envisaged in functional cure approaches when compared to an ART regimen. As mentioned before, HIV-1 transcription is a complex process that involves multiple factors; thus, several targets can be explored for therapeutic purposes.

Regulation of gene expression consists of turning genes on (gene expression) or off (gene repression) in response to the surrounding environment. Every cell transcribes just a small fraction of its genes and the remaining genes are normally turned off or epigenetically repressed, conserving energy and resources [110]. Turning on a gene requires a significant amount of energy; therefore, gene repression is, in theory, simpler to achieve. Indeed, silencing approaches have become a focus of research for HIV-1 functional cure approaches.

**Figure 3:**
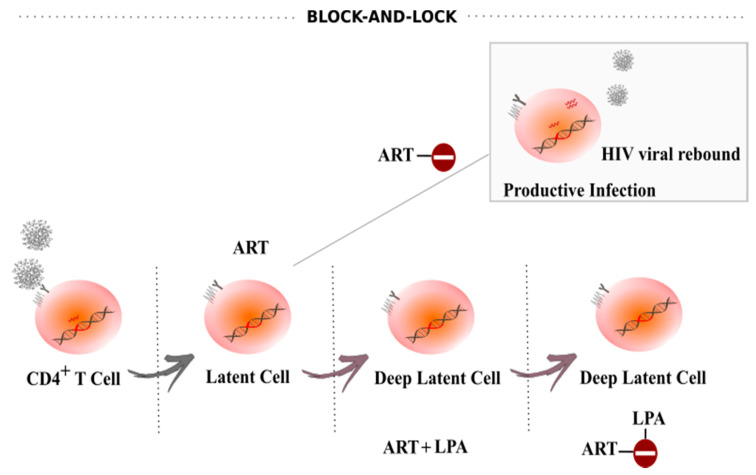
Block-and-lock approach as a functional cure for HIV-1 infection. The block-and-lock approach entails the long-term durable silencing of viral gene expression. Supplementation of ART with a latency promoting agent (LPA), such as the Tat inhibitor didehydro-Cortistatin A (dCA), could suppress ongoing transcriptional events, induce epigenetic silencing over time and promote a state of “deep latency”, blocking or limiting viral rebound upon treatment interruption.

The block-and-lock approach relies on targeting either HIV-1 or host-specific factors to induce a state of deep and irreversible latency in the absence of ART (Figure 3) [109,111]. Ideally, deep latency observed in some elite controllers could be therapeutically mimicked, ultimately providing a drug-free HIV-1 remission. Here, we discuss a few of the approaches explored towards this goal.

### 5.1. Tat

Tat is a small 14 kDa protein that potently activates HIV-1 gene expression. Tat protein is considered a desirable target for drug development because it is expressed early on during infection [46,55], it is highly conserved among HIV-1 isolates [112], it has no cellular homolog, and, finally, its direct inhibition blocks the feedback loop that drives exponential viral production [113]. Although several compounds have been previously tested against Tat or its interactions (TAR-Tat-P-TEFb), up until recently, these compounds showed low specificity or poor pharmacokinetics, and none has reached the clinic [114].

Our group first described didehydro-Cortistatin A (dCA) in 2012, an equipotent analog of the natural product Cortistatin A (CA), as a specific and potent Tat inhibitor. dCA binds to the TAR-binding domain of Tat, the basic domain, blocking transcriptional elongation of the HIV-1 promoter, and consequently leading to the epigenetic silencing of the HIV-1 promoter [115]. In acutely and chronically infected cells, as well as primary CD4^+^ T cells, we demonstrated transcriptional inhibition of HIV-1 to undetectable or very low levels of viral RNA, using subnanomolar concentrations of dCA, without cell-associated toxicity [115]. Of note, dCA inhibits both HIV-1, HIV-2, and SIV Tat-mediated transcription from the viral promoter [116], given the conservation of these protein’s basic region. Importantly, dCA does not interact with the cellular protein HEXIM that has a similar but structurally different basic region [117]. Using primary CD4^+^ T cells isolated from ART-suppressed individuals, we showed that long-term dCA treatment strongly suppresses viral transcription, eventually driving a state of persistent latency that is refractory to reactivation with LRAs. Importantly, discontinuation of dCA and ART treatment maintains viral suppression, suggesting long-lasting effects [118]. In agreement, our in vivo studies using the humanized bone marrow-liver-thymus (BLT) mouse model for HIV-1 latency, revealed that dCA treatment decreases viral RNA in tissues and delays viral rebound upon ART interruption [111]. Long-term dCA treatment does not alter the classic nucleosome position at the LTR promoter. Instead, a tighter nucleosome/DNA association is observed, which correlates with increased deacetylated histone 3 (H3) occupancy at Nuc-1. This repressive state of chromatin structure at Nuc-1 prevents RNAPII recruitment and elongation, even with stimulation with LRAs [119]. Low levels of PBAF complex is observed at the HIV promoter with dCA treatment, which is crucial for Nuc-1 remodeling and Tat-activated transcription, while the BAF complex is increased. Our results suggest that dCA accelerates the establishment of latency and, importantly, that dCA activity is directly correlated with Tat-TAR competent proviruses with no apparent off-target effects [119]. Importantly, our group reported that when intraperitoneally injected in mice, dCA crosses the blood–brain barrier (BBB) and is detected at high levels in the brain, where microglial cells are proposed to serve as an HIV reservoir. Moreover, this study demonstrated that dCA inhibits different HIV-1 clades and decrease extracellular Tat uptake by glial cell lines, which may reduce HIV-1-related neuropathogenesis [120]. Tat has been shown to potentiate cocaine-mediated reward mechanisms through disfunction of the dopaminergic system [121]. Using Tat transgenic mice, we also demonstrated the ability of dCA to reduce Tat potentiation of cocaine-mediated reward mechanisms [120].

In sum, our studies using dCA as a latency promoting agent (LPA) highlight the potential of Tat inhibitors for use in block-and-lock approaches. Adding Tat inhibitors to an ART regiment may further reduce cell-to-cell transmission, viral reactivation, and spontaneous blips, as well as drive the virus into a state of deep latency. The expectation is that long lasting transcriptional suppression may lead to prolonged or permanent epigenetic silencing of the provirus, allowing safe interruption of therapy. These studies are currently underway.

Other inhibitors of Tat-mediated transcription have been explored, namely, Triptolide (TPL), a diterpenoid epoxide isolated from *Tripterygium wilfordii* Hook F (TwHF), a natural product used for the treatment of rheumatoid arthritis [122]. TPL has shown anti-HIV-1 activity by blocking Tat function at nanomolar concentrations [123]. TPL enhances the proteasomal degradation of Tat and, consequently, the in vitro suppression of viral transcription [123]. However, the clinical potential of TPL has been debated. Wang et al. discovered that TPL induces proteasome-dependent degradation of RNAPII, inhibiting global gene transcription [124]. Another study demonstrated that TPL covalently binds to xeroderma pigmentosum group B (XPB), a subunit of the transcription factor TFIIH, and inhibits its ATPase activity, which blocks RNAPII-mediated transcription initiation [125]. Thus, due to its global inhibition of transcription, probably by interfering with important cellular functions, its clinical application may be limited by safety concerns.

Recently, a screen of an FDA-approved compound library identified Levosimendan as a potential LPA [126]. Levosimendan normally is used for the treatment of acutely decompensated heart failure [127]. Hayashi et al. discovered that Levosimendan blocks HIV-1 Tat-LTR mediated transcription. Using a PI3K inhibitor, 3-MA, they were able to overcome the inhibitory effect of levosimendan in a dose-dependent manner, suggesting that this compound is possibly involved in the Akt/PI3K pathway to inhibit HIV-1 transcription [126]. However, the specific mechanism by which this compound mediates the inhibition of HIV-1 transcription and reactivation is still under investigation and needs to be further elucidated. Additionally, they showed that it also suppresses HIV-1 reactivation from latency, using several HIV-1 latency cell lines, primary CD4^+^ T cell models of HIV-1 latency, and primary CD4^+^ T cells isolated from HIV-1-infected individuals on ART. On the same screen, Hayashi et al. also found Spironolactone (SP) as another anti-HIV-1 agent [126]. SP can promote the degradation of the XPB subunit of TFIIH [128] and it was shown to inhibit acute HIV-1 infection of cell lines and primary CD4^+^ T cells by blocking HIV-1 transcription [129]. Recently, our group demonstrated that long-term SP treatment rapidly reduces ongoing transcription in latently infected cell line models in what appears to be a Tat-TAR independent mechanism and was associated with a reduction in RNAPII recruitment to the HIV-1 genome (Mori, L. et al., in press). SP treatment potently reduced HIV-1 reactivation with exposure to a range of LRAs and, importantly, blocked HIV-1 reactivation in ex vivo stimulated primary CD4^+^ T cells. Unfortunately, SP inhibition of HIV-1 transcription was not long lived, with viral rebound occurring rapidly upon treatment interruption and XPB replenishment. It will be important to assess the effects of SP alongside other longer-lasting LPA, such as dCA, for example. Since both SP and Levosimendan are FDA-approved compounds, it may accelerate their investigation in humans.

### 5.2. Facilitates Chromatin Transcription (FACT) Complex

The FACT protein complex is a histone chaperone responsible for the removal of H2A/H2B dimer to facilitate RNAPII-driven transcription by destabilizing the structure of nucleosome and depositing core histones back afterward. It was demonstrated that this host FACT complex (SUPT16H and SSRP1) restricts HIV-1 replication through transcriptional suppression [130]. Curaxins are small molecule compounds that target the activity of the FACT complex and have been successfully tested for their anti-tumor activity [131]. Maxime et al. showed that curaxin CBL0100 could also inhibit HIV-1 through FACT targeting. It was proposed that CBL0100 targets HIV-1 transcription elongation by preventing FACT-induced nucleosome disassembly and RNAPII recruitment to Nuc-1, independent of the NF-κB pathway. Results showed that CBL0100 alone moderately blocks acute infection and inhibits HIV-1 reactivation by LRAs, and intensifies the activity of ART. Therefore, CBL0100 in combination with ART could promote faster viral clearance, and alone or in combination with other LPAs could block HIV-1 reactivation and reduce the HIV-1 viral reservoir under ART [132].

Studies are needed to fully understand the role of this complex in HIV-1 latency establishment, maintenance, and reactivation. However, CBL0100 could be a potential target as LPA for functional cure approaches.

### 5.3. mTOR Complex (mTORC)

mTOR, a serine/threonine kinase that forms two complexes, mTOR1 and mTOR2, is involved in a variety of cellular processes, such as the regulation of glucose metabolism, cell growth, energy balance and viability [133,134]. Rapamycin is a bacterial macrolide used for the treatment of renal transplantation rejection and is an allosteric inhibitor of mTOR kinase, that selectively inhibits mTORC1. Heredia et al. demonstrated that rapamycin can inhibit HIV-1 replication in vitro by down-regulation of CCR5 expression, the HIV-1 major co-receptor [135]. Later, the same team demonstrated that INK128, an inhibitor of both mTORC1 and mTORC2, successfully suppressed HIV-1 replication in vivo [136]. INK128 inhibited both basal and induced (by PMA) HIV-1 transcription [136]. Inhibition of HIV-1 transcription is consistent with inhibition of mTORC2, which is essential for phosphorylation of PKC isoforms [137], required for NF-κB induction of HIV-1 transcription [138]. Later, in 2017, Besnard et al. supported the idea that the inhibition of mTORC1 and mTORC2 can suppress HIV-1 reactivation. Through a human genome-wide shRNA screen, the authors uncover the mTOR pathway as a modulator of HIV-1 latency. Results showed that mTORC1 and mTORC2 inhibition strongly suppress reactivation of HIV-1 in a CD4^+^ T cell line model and in HIV-1-infected patient cells. MLST8 knockdown, a subunit shared by mTORC1 and mTORC2, suppressed HIV-1 reactivation under PMA stimulation, but not upon BET and HDAC inhibitor treatment. This supports the role that PKC-dependent NF-κB activation seems to be an important target of mTOR and its relation to HIV-1 latency [139]. In another study, Ji Shan et al. adapted a genome-wide CRISPR screening approach in a T-cell-based latency model and discovered that TSC1 and DEPDC5, two mTORC1 natural inhibitory genes, are potentially involved in HIV-1 latency. Their results suggested that TSC1 and DEPDC5 suppress the AKT-mTORC1 pathway activity and hamper the initiation of HIV-1 transcriptional translation to maintain latency [140]. In 2019, two clinical trials, NCT02990312 and NCT02440789, were initiated to evaluate the impact of rapamycin on HIV-1 persistence and immune activation/inflammation.

Targeting cellular proteins could be an attractive approach to overcome HIV-1 drug resistance. However, the mTOR pathway controls key cellular processes and inhibitors may come with some off-target activity. The feedback from clinical trials will determine their future clinical use.

### 5.4. Bromodomain-Containing Protein 4 (BRD4)

BRD4 is a member of the bromodomain and extra terminal domain (BET) family and is involved in the regulation of gene expression [77,78]. As already mentioned in Section 3.1, BRD4 acts positively or negatively on the regulation of HIV-1 transcription [50,75,76,78]. Through a structure-guided drug design, Niu Qingli et al. identified a small molecule, ZL0580, that selectively binds to BRD4 and induces epigenetic HIV-1 suppression using in vitro and ex vivo models. The results revealed that ZL0580 accelerates HIV-1 suppression in combination with ART and delayed viral rebound upon ART interruption ex vivo. ZL0580 binds to BRD4 and inhibits Tat transactivation and transcription elongation. Specifically, ZL0580 decreases the binding of P-TEFb (CDK9) to Tat, while enhances BRD4-CDK9 biding and reduces Tat biding to LTR. Additionally, ZL0580 induces a repressive chromatin structure at the HIV-1 promoter [141].

Despite promising results, further evaluation is needed to elucidate the in vivo effects of ZL0580. It should be noted that viral rebound eventually occurs after drug removal, which indicates a potential limitation for ZL0580, as well as for BRD4 inhibition, that cannot permanently or durably suppress latent viruses. BRD4 is functionally versatile and thus more research to understand the consequences of its inhibition is needed.

### 5.5. Heat Shock Protein 90 (HSP90)

HSP90 is a heat-shock chaperone that localizes at the HIV-1 promoter and regulates its expression [142]. Joshi et al. demonstrated that the cytosolic HSP90 isoform is an essential HIV-1 host factor, and its inhibition by RNA interference blocks HIV-1 replication in primary human T cells [143]. HSP90 was shown to be involved in HIV-1 reactivation through modulation of the NF-κB signaling pathway and stimulation of Tat-mediated HIV-1 transcription [144,145].

An in vivo study using Hsp90 inhibitors (AUY922 or 17-AAG) reported that viral rebound was blocked up to 11 weeks after treatment interruption in mice pretreated with a reverse transcriptase inhibitor (EFdA) plus AUY922 or 17-AAG [146]. Infectious viruses were successfully recovered by heat shock or cell activation from PBMCs and spleen, suggesting that these cells were latently infected [146].

GV1001, an MHC class II-restricted peptide vaccine designed to induce T-cell immunity to telomerase, can reduce the levels of HSP90 inside the cell and on the cell surface [147]. It was previously shown that GV1001 suppressed the replication of the Hepatitis C virus (HCV) through the interaction with HSP90 [148]. Kim et al., likewise, reported that GV1001 can significantly suppress HIV-1 replication, as well as viral reactivation from latently infected cells. Of note, GV1001 suppressed NF-κB activation and, consequently, downregulated Tat-dependent transcriptional activity and HIV-1 RNA production [144]. These results suggested that HIV-1 suppression by GV1001 is HSP90-dependent, since treatment with an anti-HSP antibody resulted in the loss of compound activity.

It seems clear that HSP90 is implicated in NF-κB activity [145]. Investigating the underlying molecular mechanism of NF-κB suppression is needed. Together, these data suggest that HSP90 inhibitors can also be studied as potential LPAs to achieve a functional HIV-1 cure.

## 6. Other Sterilizing/Functional Cure Strategies

### 6.1. RNA-Based Strategies

miRNAs are a class of small single-stranded non-coding sequences that control gene expression by translational inhibition or mRNA degradation [149]. Several studies suggest a role of miRNAs in the regulation of HIV-1 transcription and, therefore, have been explored to fight HIV-1 infection. In primary resting CD4^+^ T cells, cellular miRNAs, such as miR-28, miR-125b, miR-150, miR-223, and miR-382, potently inhibit HIV-1 expression through interaction with the 3´end of the HIV-1 mRNA, promoting HIV-1 latency. The transfection of corresponding antagomiRs reactivated the virus, suggesting that these miRNAs can be targeted as a therapeutic approach to reactivate HIV-1 from latency [150]. Similarly, cellular miRNAs against Tat or its interactions (TAR-Tat-P-TEFb complex), such as miR-198 and miR-27b, have been studied as regulators of cyclin T1 protein levels [151,152]. Regarding viral mRNAs, HIV-1 generates miR-TAR-3p that might serve as a negative suppressor of HIV-1 replication in primary human monocyte-derived macrophages, possibly by targeting the TAR element in the 5′-LTR, and thus inhibiting HIV-1 genome transcription [153]. Together, these miRNAs can be explored as targets for suppression or activation of HIV-1 replication.

**Figure 4:**
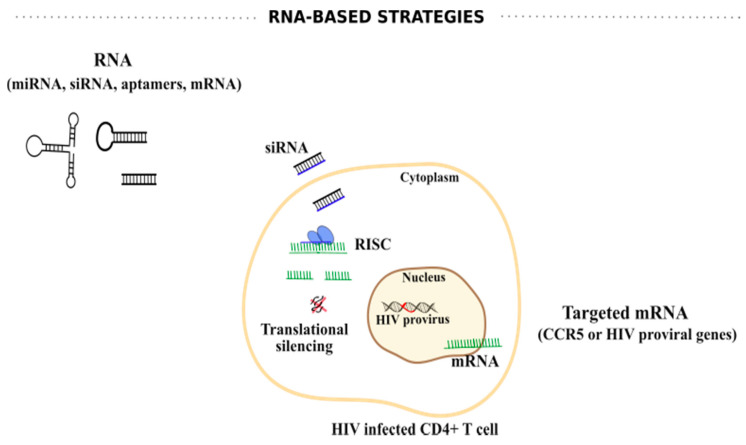
RNA-based strategies against HIV infection. RNA-based strategies include the use of miRNAs, siRNAs, RNA aptamers, and mRNAs. These RNAs can be synthesized to specifically bind to CCR5, the major co-receptor for HIV entry, or bind to crucial regions into HIV proviral genome. siRNAs assemble with cellular host proteins to form an RNA-induced silencing complex (RISC). Only one strand (guide) is maintained and produces an active RISC that triggers the targeted mRNA degradation, culminating in translational silencing.

Given the role of RNAs on HIV-1 replication, other RNA-based strategies including anti-sense RNAs, small interfering RNAs (siRNAs) and aptamers, among others, have been explored to target HIV-1 transcription (Figure 4). For example, an antisense RNA sequence against the envelope region of HIV-1 expressed by an HIV-1-based lentiviral vector, VRX494, demonstrated inhibition of different HIV-1 strains at a clinically relevant multiplicity of infection (MOI) with efficient gene delivery to primary lymphocytes [154]. Additionally, anti-HIV-1 aptamers have also demonstrated some potential at suppressing HIV-1 infection [155,156]. Zhou et al. constructed a dual functioning anti-gp120 aptamer-siRNA chimera with potent anti-HIV-1 activities in vitro, as well as in vivo [157]. The data demonstrated that this aptamer-siRNA chimera is internalized by cells expressing HIV-1 gp120, and anti-tat/rev siRNA is released and inhibits HIV-1 replication [157]. Similarly, efficient delivery of anti-protease or anti-CCR5 aptamer-based siRNA demonstrated gene knockdown with consequent HIV-1 suppression [158,159]. Despite the potential of aptamers, challenges such as pharmacokinetics, bioavailability, biodistribution, stability in serum, and non-specific immune stimulation still need to be addressed [160].

Synthetic siRNAs and short hairpin RNAs (shRNAs) have also been applied to target different crucial HIV-1-encoded RNAs, including tat, rev, gag, nef, and LTR regions [161,162,163,164,165]. However, the virus likely acquires single or multiple mutations or deletions enabling its escape [166,167]. Thus, targeting highly conserved sequences and/or combining different siRNAs or shRNAs is extremely important. For example, Naito et al., through an exhaustive computational analysis, reported highly effective siRNAs against divergent HIV-1 strains, including siRNAs with maximal conservation (>70%) against the sequences of the trans-activation response (TAR) and the polyadenylation signal (poly-A) with 84% of inhibition [168]. Another study, using shRNA targeting the R region of HIV-1 LTR in combination with an shRNA targeting CCR5 mRNA was successfully evaluated in a humanized mouse model using hematopoietic stem/progenitor cell (HSPC) gene therapy [169]. Interestingly, an ongoing clinical trial (NCT03517631) is evaluating the efficacy and safety profile of autologous CD34^+^ cells that stably express multiplexed shRNAs targeting CCR5 and the HIV-1 genome to fight HIV-1 infection.

Despite some encouraging results with RNA-based strategies, concerns arise, including the efficient delivery of these nucleic acids by shuttle vectors, namely, non-viral and viral systems, low bio-stability, their negative charge and size, difficult passage across cellular membranes, and unmodified siRNA instability in the bloodstream [170]. Therefore, continuous improvement of these shortcomings is required to achieve a promising RNA-based strategy against HIV-1.

### 6.2. CRISPR/Cas Systems

The CRISPR/Cas system is a bacterial defense mechanism against viruses that targets specific genes in nearly every organism, including plants, mice, and human cells [171]. Clinical trials have been initiated to explore the safety and feasibility of CRISPR-based therapies. Data from a recently completed clinical trial (NCT03399448), led by researchers at the University of Pennsylvania, suggest that CRISPR-Cas9 designed to engineer T cells to boost cancer-fighting capabilities is safe [172]. However, data are still outstanding from larger cohorts and longer observation periods after treatment to fully assess the safety of this approach.

**Figure 5:**
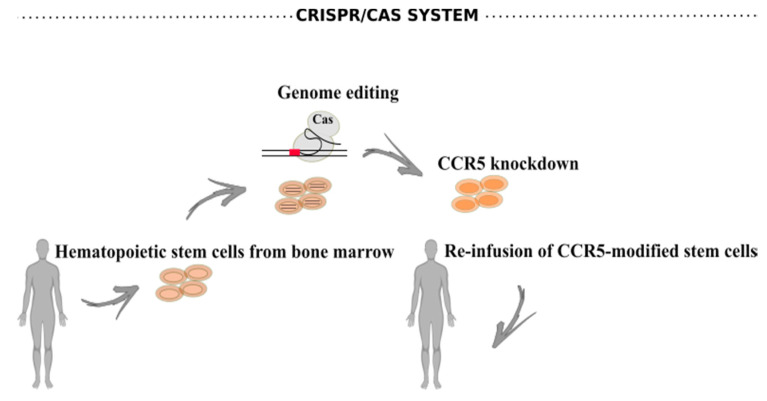
CRISPR/Cas system to generate CCR5-resistant stem cells. Hematopoietic stem cells are collected from bone marrow of infected individuals. These cells are expanded ex vivo upon genome editing by the CRISPR/Cas system targeting the CCR5 co-receptor. Subsequently, the CCR5-modified stem cells are infused back to the same individual.

CCR5 is the major HIV-1 coreceptor for transmitted viruses used early during infection [173]. The majority of genome-editing therapies applied to HIV-1 infection, in clinical trials, consist of autologous transplantation of CCR5-modified stem cells (Figure 5). These cells were previously modified and expanded ex vivo upon transduction with a viral vector expressing a zinc-finger nuclease that inactivates the CCR5 gene [174].

The CRISPR/Cas system has been exploited for disruption of crucial HIV-1 genes, as well as excision of the HIV-1 provirus from infected cells [175,176,177]. Although HIV-1 may escape CRISPR/Cas knockdown in vitro through the generation of mutations [178], the combination of single guide RNAs (sgRNAs) to multiple HIV-1 genes (multiplex genome-editing) may prevent viral escape. Multiplex genome-editing may thus become an effective strategy for the cure of HIV-1 infection [179,180]. Like antiretroviral drug regimens, gene therapy against multiple viral targets might be required to achieve significant clinical benefit.

Thus far, only one clinical trial is ongoing with CRISPR-editing of the CCR5 gene (NCT03164135). The first results reported successful allogeneic transplantation and long-term engraftment of CRISPR-Cas9-edited with CCR5-ablated HSPCs in an HIV-1 patient with acute lymphoblastic leukemia. The clinical safety profile was higher compared to other HSPC-based gene therapies. Of note, no apparent clinical adverse or off-target effects were reported; however, CCR5 ablation was less than 8% in the genome of circulating bone marrow cells [181]. Although the proof-of-concept that transplantation and long-term engraftment of CRISPR-edited allogeneic HSPCs was achieved, further investigation is needed to improve the efficacy of gene-editing by the CRISPR/Cas system. Despite CCR5 editing suggesting exciting possibilities for an HIV-1 cure, CXCR4 also allows HIV-1 entry into the host cell and thus needs equal consideration. Indeed, it was reported that a patient exhibited tropism change from dominantly CCR5-tropic HIV-1 before stem-cell transplantation to a CXCR4-tropic HIV-1 after transplantation with stem cells homozygous for the CCR5Δ32 mutation [182].

CRISPR/Cas9 is a powerful technology; however, some obstacles, such as safety, efficacy, and specificity, must be surpassed. Ethical concerns also need the utmost consideration. Potential permanent harm in genetically modified individuals and their descendants as well as exacerbation of social inequality are some of the issues that need pondering [183,184]. In December 2018, a disturbing announcement by a Chinese researcher revealed the CCR5 gene editing of two embryos [185], which prompted social debate regarding the use of this technology. Important questions arise: Where to draw the line between disease treatment and enhancement? In regard to germline editing, do we have the right to modify future generations? What about religious objections? These and other questions should be intensely discussed and should serve as an appeal for defining international regulation and guidelines for CRISPR´s use.

## 7. Discussion

The ART treatment of HIV-1 infection undoubtedly improves the quality of life of individuals living with HIV-1 and decreases HIV-1 incidence worldwide. However, financial costs, distribution, and stigma remain unsolved. Despite all efforts, thus far there are no safe, effective, scalable, and durable interventions to achieve an HIV-1 remission or cure that is feasible for everyone. Nevertheless, our improved knowledge concerning basic mechanisms of HIV-1 latency and regulation of HIV-1 transcription has opened novel therapeutic avenues of research. The shock-and-kill and block-and-lock approaches are currently being explored to tackle HIV-1 latency. Additionally, gene-editing tools have seen an immense development with the CRISPR/Cas system, demonstrating exciting results in vitro and ex vivo. However, when translated to in vivo, some challenges remain, such as delivery and off-target effects, which have been hampering its clinical success.

The block-and-lock approach, as a way of functionally curing HIV-1, if successful, would provide a long-term remission from HIV-1, even in the absence of ART. The proof-of-concept of this approach came with the discovery of dCA. dCA potently binds Tat protein from different HIV-1 strains and efficiently disrupts the Tat/TAR axis [115,118,119], suppressing HIV-1 transcription and replication. Long-term dCA treatment results in a long lasting heterochromatization of the HIV-1 promoter locus [119]. Moreover, in in vivo models of HIV-1 infection and latency, the results demonstrate a delay in viral rebound upon treatment interruption. Ideally, the combinatory treatment of dCA with ART would delay viral transcription, reactivation, and prevent the renewal of the latent reservoir. In parallel, it would likely have the added benefit of reducing chronic immune activation and inflammation. This is particularly important since it contributes to HIV-1 persistence through different mechanisms, such as the stimulation of infected cells to produce virions, inducing proliferation of infected cells, and inhibiting HIV-1-specific clearance mechanisms through immune exhaustion, reviewed by N. Klatt et al. [186].

Like dCA, we described here other compounds such as Triptolide, Levosimendan, and Spironolactone, that affect HIV-1 transcription, and thus disrupt not only the replication-competent but also the defective proviruses. However, further evaluation in vivo is needed to validate these compounds. Host factors can also be targeted to inhibit HIV-1 latency reversal. Compounds such as curaxin, Triptolide, or HSP90 inhibitors (AUY922 or 17-AAG) have been evaluated, but the clinical safety is still scarce or discouraging. Moreover, they all harbor inherent pleiotropic consequences and are subject to variability in response, highlighting the potential of using Tat- or viral specific targeting compounds.

Finally, it is our belief that it is probably easier to reinforce latency to control HIV-1 rather than purging the viral reservoir (shock-and-kill) [187]. Indeed, 5–8% of the human genome contains sequences that are originally from retrovirus that no longer express or produce productive and infectious virions. Therefore, a successful block-and-lock approach could potentially turn an integrated HIV-1 provirus into another harmless endogenous retrovirus incapable of replication [188,189]. We suggest that small molecule compounds such as dCA can be an affordable and scalable way to achieve an HIV-1 remission. Moreover, we want to reinforce the notion that targeting a viral protein, such as Tat, provides a specific and direct inhibitory effect, which, compared with host-targeted approaches, may have unwanted off-target activities.

As with any novel developing therapeutic approach, further investigation is needed to understand the full clinical potential of the block-and-lock approach. For instance: (1) What is the impact of LPAs treatment on reservoir size? (2) How durable is the repressive heterochromatin environment at the silenced HIV promoter? (3) What are the mechanisms of viral resistance to LPAs? (4) What is the impact of LPAs on immune activation and chronic inflammation? All these questions need addressing to move the approach forward.

In sum, block-and-lock approaches are showing exciting potential and efforts have been made to move forward to the clinic, even if a considerable amount of work is still outstanding. A combination of different approaches may also be envisaged, for instance, using the shock-and-kill approach to an efficient clearance of easily reactivated virus followed by the use of LPAs to silence the remaining proviruses that might be more susceptible to transcriptional silencing and epigenetic suppression.

With the improved long-acting ART, HIV-1 prevention strategies, and the latest advances in HIV-1 cure strategies, we are reaching a turning point for cautious optimism for HIV-1 therapy in the next decade.
